# Intrathecal dexmedetomidine can decrease the 95% effective dose of bupivacaine in spinal anesthesia for cesarean section: A prospective, double-blinded, randomized study

**DOI:** 10.1097/MD.0000000000014666

**Published:** 2019-03-01

**Authors:** Lin Liu, Jing Qian, Bei Shen, Fei Xiao, Huaxiang Shen

**Affiliations:** a Department of Anesthesia; b Department of Obstetrics, Jiaxing University Affiliated Women and Children Hospital, Jiaxing City, China.

**Keywords:** bupivacaine, cesarean section, dexmedetomidine, dose–response, intrathecal

## Abstract

**Background:**

Dexmedetomidine (Dex), as an adjuvant, has been reported to prolong the duration of spinal analgesia when adding to local anesthetic. We hypothesized that Dex could enhance the efficiency of intrathecal bupivacaine for spinal anesthesia in cesarean section. The aim of his study is to test our hypothesis that 5 μg Dex could enhance the efficiency of intrathecal bupivacaine and reduce the dose requirement of spinal bupivacaine for patients undergoing cesarean section.

**Methods:**

Ninety patients with ASA I or II, who underwent cesarean section, were randomized into 2 groups: group D (bupivacaine + 5 μg Dex) and group C (bupivacaine + the same volume of saline). The subsequent dose of spinal bupivacaine was determined by the improved up–down allocation method. The initial dose of bupivacaine in the 2 groups was 4 mg, and the subsequent dose for the following patient was depended on the probability of the current dose. ED95 of spinal bupivacaine was calculated using logistic regression model.

**Results:**

The ED95 and 95% confidence intervals (95% CI) of spinal hyperbaric bupivacaine in group D and group C were 7.4 mg (95% CI, 5.6–12.4 mg) and 11.0 mg (95% CI, 4.4–56.8 mg), respectively. The duration of sensory block was 120.5 ± 37.0 minutes in Dex group and 70.5 ± 34.5 minutes in Control group, respectively (*P* < .05). The duration of analgesia was 230.5 ± 40.5 minutes in Dex group and 145.1 ± 28.5 minutes in Control group, respectively (*P* < .001). The consumption of postoperative rescued sufentanil was significantly lower in Dex group than in the Control group (56.3 ± 9.4 vs 65.9 ± 10.7 μg). There was not significantly different in the patient satisfaction of analgesia, incidence of side effects, neonatal outcomes and neurological deficit between the 2 groups.

**Conclusion:**

Intrathecal 5 μg Dex enhances the efficacy of spinal bupivacaine by 24% in patients undergoing cesarean section with spinal anesthesia. No additional side effect was observed by adding spinal Dex.

## 1 Introduction

Spinal anesthesia is widely used in cesarean section, but it is associated with high incidence of hypotension.^[1]^ To reduce the occurrence of hypotension, intrathecal adjuvant was recommended to use in spinal anesthesia, with the aim in reducing the dose of intrathecal local anesthetic, which can subsequently decrease the incidence of spinal-induced hypotension.^[2–4]^ Recently, dexmedetomidine (Dex) has been assessed in central and peripheral nerve block, and found that it has the potential to prolong the duration of analgesia. We suspected that Dex may also have the ability to enhance the efficiency of intrathecal bupivacaine for spinal anesthesia in cesarean section. The aim of this study is to verify our hypothesis that Dex can enhance the efficiency of intrathecal bupivacaine with an improved up–down allocation method.

## 2 Methods

### 2.1 Design

We conducted a prospective, double-blinded and randomized study to calculate the ED95 of intrathecal hyperbaric bupivacaine, combined with or without the adjuvant of 5 mcg of Dex, for patients undergoing cesarean section in spinal anesthesia.

### 2.2 Participants and setting

Following the Institutional Ethics Committee of Jiaxing University Affiliated Women and Children Hospital approval and patient written informed consent from all patients, 90 parturients with the statue of American Society of Anesthesiologists’ physical class I or II, prepared for an elective cesarean section, were recruited in the current clinical trial. Exclusion criteria were as follows: BMI >35 kg/m^2^, gestational age <37 weeks, diabetes or gestational diabetes, hypertension or pre-eclampsia, contraindications to intraspinal anesthesia. The current clinical trial was registered in Chinese Clinical Trial Registry by Dr. F. X. and getting a number of ChiCTR-TRC-14004954.

According to a random number list generated via a software (Microsoft, Excel) and kept in a sealed opaque envelope before the study initialed, patients were distributed into group C (control group) and group D (Dex group), and there were 45 patients in each group.

There were no premedication. When patients arrived in operating room, an 18-G intravenous cannula was inserted into the peripheric vein and 37° C *Lactate Ringer's solutions* were injected just to keep the vein open before spinal anesthesia. Then the basic monitoring, such as electrocardiogram (ECG), non-invasive blood pressure (NIBP), and oxygen saturation (SpO_2_), was measured and studied. The average value of the first 3 readings was considered as the basal NIBP and HR.

With the parturients in left lateral position, the combined spinal–epidural anesthesia was performed using the needle-through-needle technique. In details, an 18-G Tuohy needle was penetrated into epidural space at L_3–4_ interspace using loss-of-resistance-to-air technique (air volume was <2 mL). Then a pencil-tip 27 G spinal needle pierced the endorhachis through the Tuohy needle. Before removing the spinal needle, withdrawing the CSF again making sure the drug was injected in the subarachnoid space (if failed to withdraw the CSF, the subject was excluded from the study). After spinal injection, an epidural catheter was placed into epidural space with 3 –4 cm and no local agent delivered at this moment. Patients were then in supine position with a 15-degree wedge under right buttock. At the same time of spinal injection, 5 mL/kg 37°C *Lactate Ringer's solutions* were co-loaded in 20–30 minutes.

The study drug was prepared in an unsterile condition by an anesthetist (J.Q.), who was not involved in assessing the following dose of bupivacaine, which will be delivered by another attending anesthetist (L.L. and B.S.) who kept blinded to the study drug. The mixed bupivacaine for patients in the 2 groups is as follows: varied dose of hyperbaric bupivacaine in group C and varied dose of hyperbaric bupivacaine combined with 5 μg Dex (Jiangsu Hengrui Medical Company, LTD China; Production batch: 16090232.) in group D. And the study drug mixed with 0.5 mL of 10% dextrose, and diluted into 3 mL with saline.

The subsequent dose of spinal bupivacaine was determined by the improved up–down allocation method.^[5]^ The initial dose of bupivacaine in the 2 groups received by the first patient was 4 mg. The subsequent dose for the following patient was depended on the probability of the current dose. If the probability of the current dose exceeded 95%, the following intrathecal dose of bupivacaine will decrease 1 mg compared with the current dose. On the contrary, the following intrathecal dose of bupivacaine will increase 1 mg, if the probability of the current dose is <95%. And if the probability still remained 95%, the following dose of intrathecal bupivacaine was not altered for the following patient. According to our prior study, if a sensory block level (loss of pinprick) ≥T6 achieved in 10 minutes after spinal injection and no epidural supplement was needed during surgery, it was regarded as an effective dose. Otherwise, it was considered as an ineffective dose. An ineffective dose will rescue by 5 mL of 2% lidocaine via epidural catheter at 5 minutes intervals till the sensory block level reached at T6 at least.

### 2.3 Measurements

The primary outcome of this study was the effective or ineffective dose of spinal anesthesia. The secondary outcomes of this study were the characteristics of spinal anesthesia and duration of spinal analgesia and side effects.

HR and NIBP were measured at 1-minute intervals at the first 10 minutes after intrathecal injection, subsequently at 5-minute interval till the surgery completed. A descent in SBP ≥20% baseline SBP or SBP <90 mm Hg was regarded as hypotension and treated with 100 μg of phenylephrine. HR <55 bpm was regarded as bradycardia and 0.5 mg of atropine was intravenously delivered.

Via a blunt 17-G needle, sensory block level was gently checked along the mid-clavicular. The patient was required to inform the pain sensation of “hurt” or not. If the sensory block level was not consistent, the lower side was regarded as the sensory block level. The duration from spinal injection to a T10 sensory block achieved was considered as onset time of spinal block. The period from the onset time to 2-segment regression was regarded as duration of sensory block. Motor block was assessed and graded using Bromage Score^[6]^: 0 = no block, 1 = can bend knee and ankles, 2 = can only bend ankles, 3 = cannot bend ankles). The duration from spinal injection to Bromage Score of 1 being reached was defined as the onset time of motor block. The interval time from intrathecal injection to the first time of postoperative rescued requirement of analgesia was regarded as duration of spinal anesthesia. Postoperative pain was rescued by a setting of patient-controlled intravenous analgesia (PCIA) pump. And it was set with a bolus of 2 μg sufentanil and 10 minutes of locking time and no background dose. The characteristics of spinal anesthesia were recorded at 1-minute intervals in the first 10 minutes after intrathecal injection, 5-minute intervals during the rest time of surgery, and 30-minute intervals in the obstetric ward till the patient fully recovers. Patients’ satisfaction was graded as 1 = excellent, 2 = good, and 3 = bad.

Side effects of spinal anesthesia (such as hypotension, bradycardia, nausea and vomiting, shivering and pruritus) were studied. SpO2 <90% or breath rate <12 bpm, which was regarded as respiratory depression, was also studied. Sedation was graded as none = awake and alert, mild = awake but drowsy, moderate = asleep but arousable, severe = not arousable. Neurological deficits were also observed in the period of hospitalization and 1 month after surgery by telephone follow-up.

### 2.4 Statistical analysis

ED95 of intrathecal bupivacaine was calculated using logistic regression model. Kolmogorov–Smirnov test was used to estimate the normal distribution. Normal distributed measurement data such as demographic data and time of the characteristics of spinal anesthesia were presented as count or mean ± SD and were analyzed via Student *t* test. Mann–Whitney *U* test was used to test the difference of non-normally distributed continuous data. Statistical analysis was performed by using Graphpad Prism 5 (Version 5.01). A *P* < .05 (2-sided) was considered as statistical significance.

### 2.5 Sample size

A sample size of 45 patients for each group was determined, as under the assumed model it gave essentially bias-free estimates of the ED95 with a standard error of approximately 1 mg, which is sufficient precision to guide clinical practice.

## 3 Results

**Figure 1:**
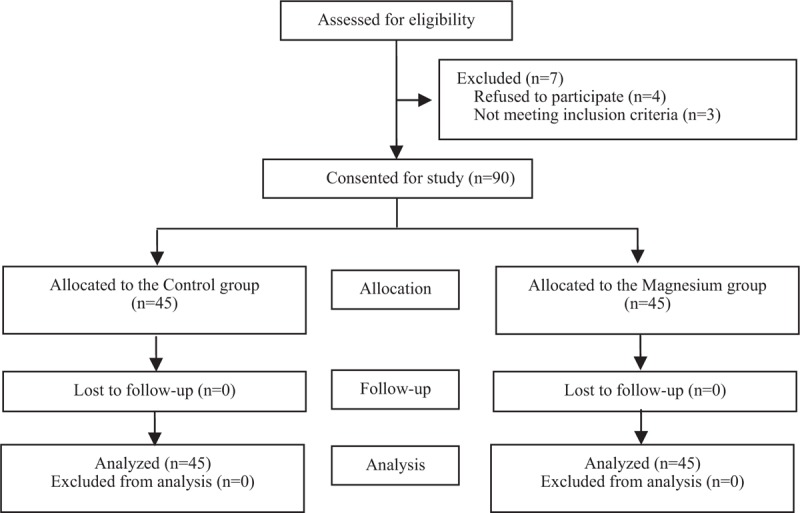
Consort diagram.

The CONSORT diagram of the present study is shown in Figure 1. Ninety-six parturients were assessed for eligibility in this study. Among them 90 parturients were randomly allocated into group D and group C. And all the 90 parturients finished the final analysis.

No significant difference in demographic, obstetric data, and duration of surgery was existed between the 2 groups (Table 1).

**Table 1:**
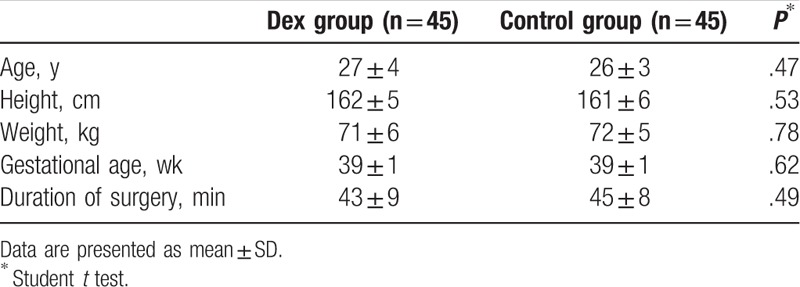
Patient's demographic, obstetric and surgical data.

**Figure 2:**
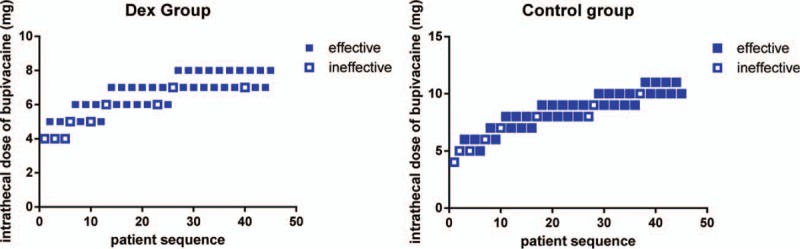
Individual response to intrathecal hyperbaric bupivacaine at corresponding dose. Unfilled square (□) represents an ineffective anesthesia to the corresponding dose of intrathecal bupivacaine for spinal anesthesia. Filled square (▪) represents an effective response to the corresponding dose of intrathecal bupivacaine for spinal anesthesia.

**Figure 3:**
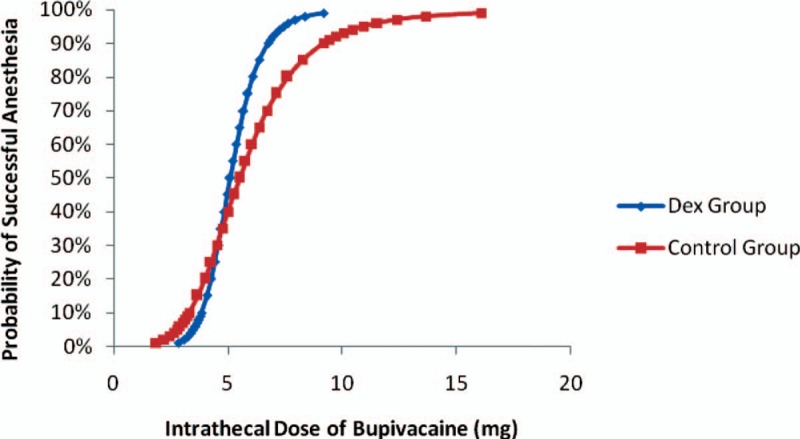
Logistic regression plot of probability of successful spinal anesthesia *versus* intrathecal bupivacaine dose of the 2 groups. Probability of 95% was used for deriving the ED95 of intrathecal bupivacaine to achieve successful spinal anesthesia for C-section. The ED95 and 95% confidence interval (CI) of intrathecal hyperbaric bupivacaine in Dex group and Control group were 7.4 mg (95% CI, 5.6–12.4 mg) and 11.0 mg (95% CI, 4.4–56.8 mg), respectively.

The ED95 and 95% confidence interval (CI) was lower in group D than in group C (7.4 mg [95% CI, 5.6–12.4) vs 11.0 mg [95% CI, 4.4–56.8 mg]). Intrathecal 5 μg Dex enhances the efficiency of spinal bupivacaine by 24%. The individual responses (effective or ineffective) to the corresponding intrathecal hyperbaric bupivacaine dose are shown in Figure 2. The dose–response curves derive from the logistic model were shown in Figure 3.

Sensory and motor block of spinal anesthesia in patients with “effective dose” were shown in Table 2. The onset time of sensory block was similar between the 2 groups (4.7 ± 1.1 vs 4.5 ± 1.3 minutes, *P* > .05). There was no significantly different in onset time to motor block between the 2 groups (4.1 ± 2.0 vs 4.3 ± 1.9, *P* > .05). The highest block level (T5 [T3–T6] vs T5 [T3-T6], *P* > .05) and the time to the highest block level (15.6 ± 4.5 vs 14.8 ± 4.2, *P* > .05) were similar between the 2 groups. There was significant difference in the duration of sensory block between group D and group C (120.5 ± 37.0 vs 70.5 ± 34.5, *P* < .05). The duration of spinal analgesia was more extended in Dex group than in Control group (230.5 ± 40.5 vs 145.1 ± 28.5, *P* < .001). The total requirement of postoperative rescued sufentanil in Dex group was lower than in Control group (56.3 ± 9.4 vs 65.9 ± 10.7, *P* < .05). There was not significantly different in the patient satisfaction of analgesia between the 2 groups (*P* > .05).

**Table 2:**
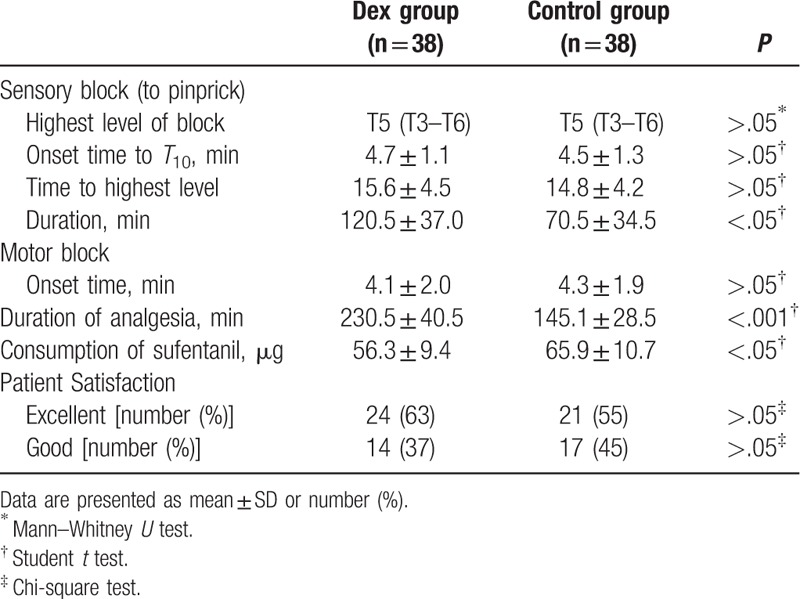
Characteristics of spinal anesthesia in patients with effective anesthesia.

There were no differences in the incidence of side effects, neonatal outcomes, and neurological deficit between the 2 groups (Table 3).

**Table 3:**
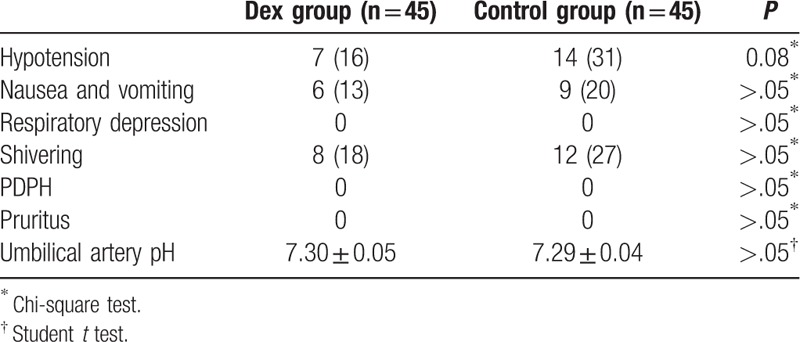
Side effects of anesthesia and neonatal outcomes.

## 4 Discussion

In the present study, we demonstrated the ED95 of intrathecal bupivacaine was 7.4 mg (95% CI, 5.6–12.4 mg) in group D and 11.0 mg (95% CI, 4.4–56.8 mg) in group C. Our results revealed that intrathecal 5 μg Dex enhances the efficiency of spinal bupivacaine by 24% in parturients undergoing cesarean section. The results in the present study also demonstrated that the duration of sensory block and analgesia was prolonged and the consumption of rescued sufentanil was reduced by adding IT 5 μg Dex. Samantaray et al^[7]^ found that the period of analgesia was prolonged and the requirement of postoperative analgesic requirement was decreased by adding 5 μg Dex to intrathecal bupivacaine, which is similar to our study. Qi et al^[8]^ also demonstrated that Dex was similar to morphine and can prolong the analgesic duration and reduce the incidence of side effect in another clinical trial.

Interestingly, there was significant difference in the ED95s between 2 groups, but the ED50s were similar as observed from the dose–response curves in Figure 3. This phenomenon may, due to the methodology of the improved up–down sequence, allocate method. The dose of intrathecal bupivacaine used in this study fluctuated around the value of ED95 not ED50. And this may result in an inconformity of statistical difference between the ED50 and ED95.

The mechanisms of Dex, which can enhance the efficiency of spinal bupivacaine, remain unknown. Perhaps it plays its role through the action of α2-AR, which subsequently induces vasoconstriction and takes its effect in this context,^[9,10]^ or directly develops its ability via α2-AR agonists rather than the action of vasoconstriction.^[11,12]^

In this study, we found that the incidence of hypotension was similar between the 2 groups (*P* = .08). Although we did not demonstrate that lowing intrathecal dose of bupivacaine can decrease the incidence of spinal-induced hypotension, there was a 15% difference of incidence of hypotension between the 2 groups, which might improve the clinical outcomes. The primary of this study was to determine the efficiency of adding Dex to intrathecal bupivacaine. Further studies could focus on this setting.

Taken into account of the safety of Dex used in spinal anesthesia, we observed the nerve deficit for a month after surgery, and found no symptoms and signs of neurological deficit were reported from the subjects. Our results suggested that intrathecal Dex is a safety adjuvant, which is consist with several studies.^[7,11,13,14]^

Limitations exist in the present study. First, there was no subjective measurement to assess the neurological deficit. Large sample size and multicenter studies should further estimate the safety of intrathecal Dex. Second, although we have demonstrated that intrathecal 5 μg Dex enhances the efficiency of spinal bupivacaine, the 95% CI of the ED95 of bupivacaine was too wide to recommend to use in clinical practice. The exact dose–response studies are needed in future.

In conclusion, intrathecal 5 μg of Dex enhances the efficiency of intrathecal bupivacaine by 24%. No additional side effects were found.

### 4.1 Other information

This work was Registration: This study was registered in a Chinese Clinical Trial Registry (ChiCTR) (registration number is ChiCTR-TRC-14004954, URL: http://www.chictr.org.cn/showproj.aspx?proj=16461).

## Acknowledgments

We thank all staffs in the Department of Anesthesia and operating room of Jiaxing Maternity and Child Care Hospital for their help in this study.

## Author contributions

**Data curation:** Jing Qian.

**Investigation:** Lin Liu, Bei Shen.

**Methodology:** Lin Liu, Jing Qian, Bei Shen, Fei Xiao, Huaxiang Shen.

**Writing – original draft:** Lin Liu, Fei Xiao, Huaxiang Shen.

**Writing – review and editing:** Lin Liu, Huaxiang Shen.

## References

[1] Gizzo S Noventa M Fagherazzi S Update on best available options in obstetrics anaesthesia: perinatal outcomes, side effects and maternal satisfaction. Fifteen years systematic literature review. Arch Gynecol Obstet 2014;290:21–34. 24659334 10.1007/s00404-014-3212-x

[2] Dyer RA Joubert IA Low-dose spinal anaesthesia for caesarean section. Curr Opin Anaesthesiol 2004;17:301–8. 17021569 10.1097/01.aco.0000137088.29861.64

[3] Xiao F Xu WP Zhang XM ED 50 and ED 95 of intrathecal bupivacaine coadministered with sufentanil for cesarean delivery under combined spinal-epidural in severely preeclamptic patients. Chin Med J (Engl) 2015;128:285–90. 25635420 10.4103/0366-6999.150083 PMC4837855

[4] Roofthooft E Van de Velde M Low-dose spinal anaesthesia for Caesarean section to prevent spinal-induced hypotension. Curr Opin Anaesthesiol 2008;21:259–62. 18458538 10.1097/ACO.0b013e3282ff5e41

[5] Ivanova A Montazer-Haghighi A Mohanty SG Improved up-and-down designs for phase I trials. Stat Med 2003;22:69–82. 12486752 10.1002/sim.1336

[6] Bromage PR A comparison of the hydrochloride and carbon dioxide salts of lidocaine and prilocaine in epidural analgesia. Acta Anaesthesiol Scand Suppl 1965;16:55–69. 5322004 10.1111/j.1399-6576.1965.tb00523.x

[7] Samantaray A Hemanth N Gunnampati K Comparison of the effects of adding dexmedetomidine versus midazolam to intrathecal bupivacaine on postoperative analgesia. Pain Physician 2015;18:71–7. 25675061

[8] Qi X Chen D Li G Comparison of intrathecal dexmedetomidine with morphine as adjuvants in cesarean sections. Biol Pharm Bull 2016;39:1455–60. 27349272 10.1248/bpb.b16-00145

[9] El-Hennawy AM Abd-Elwahab AM Abd-Elmaksoud AM Addition of clonidine or dexmedetomidine to bupivacaine prolongs caudal analgesia in children. Br J Anaesth 2009;103:268–74. 19541679 10.1093/bja/aep159

[10] Masuki S Dinenno FA Joyner MJ Selective alpha2-adrenergic properties of dexmedetomidine over clonidine in the human forearm. J Appl Physiol (1985) 2005;99:587–92. 15802370 10.1152/japplphysiol.00147.2005

[11] Eledjam JJ Deschodt J Viel EJ Brachial plexus block with bupivacaine: effects of added alpha-adrenergic agonists: comparison between clonidine and epinephrine. Can J Anaesth 1991;38:870–5. 1742820 10.1007/BF03036962

[12] Yoshitomi T Kohjitani A Maeda S Dexmedetomidine enhances the local anesthetics action of lidocaine via an alpha-2A adrenoceptor. Anesth Analg 2008;107:96–101. 18635472 10.1213/ane.0b013e318176be73

[13] Mohamed SA El-Rahman AM Fares KM Intrathecal dexmedetomidine, ketamine, and their combination added to bupivacaine for postoperative analgesia in major abdominal cancer surgery. Pain Physician 2016;19:E829–839. 27454273

[14] Gupta M Gupta P Singh DK Effect of 3 different doses of intrathecal dexmedetomidine (2.5 μg, 5 μg, and 10 μg) on subarachnoid block characteristics: a prospective randomized double blind dose-response trial. Pain Physician 2016;19:E411–420. 27008297

